# Impact of Adverse Childhood Experiences on Psychotic-Like Symptoms and Stress Reactivity in Daily Life in Nonclinical Young Adults

**DOI:** 10.1371/journal.pone.0153557

**Published:** 2016-04-15

**Authors:** Paula Cristóbal-Narváez, Tamara Sheinbaum, Sergi Ballespí, Mercè Mitjavila, Inez Myin-Germeys, Thomas R. Kwapil, Neus Barrantes-Vidal

**Affiliations:** 1 Departament de Psicologia Clínica i de la Salut, Universitat Autònoma de Barcelona, Barcelona, Spain; 2 Center for Contextual Psychiatry, Department of Neuroscience, KU Leuven, Leuven, Belgium; 3 Department of Psychology, University of North Carolina at Greensboro, Greensboro, North Carolina, United States of America; 4 Sant Pere Claver – Fundació Sanitària, Barcelona, Spain; 5 Centre for Biomedical Research Network on Mental Health (CIBERSAM), Instituto de Salud Carlos III, Madrid, Spain; Katholieke Universiteit Leuven, BELGIUM

## Abstract

**Background:**

There is increasing interest in elucidating the association of different childhood adversities with psychosis-spectrum symptoms as well as the mechanistic processes involved. This study used experience sampling methodology to examine (i) associations of a range of childhood adversities with psychosis symptom domains in daily life; (ii) whether associations of abuse and neglect with symptoms are consistent across self-report and interview methods of trauma assessment; and (iii) the role of different adversities in moderating affective, psychotic-like, and paranoid reactivity to situational and social stressors.

**Method:**

A total of 206 nonclinical young adults were administered self-report and interview measures to assess childhood abuse, neglect, bullying, losses, and general traumatic events. Participants received personal digital assistants that signaled them randomly eight times daily for one week to complete questionnaires about current experiences, including symptoms, affect, and stress.

**Results:**

Self-reported and interview-based abuse and neglect were associated with psychotic-like and paranoid symptoms, whereas only self-reported neglect was associated with negative-like symptoms. Bullying was associated with psychotic-like symptoms. Losses and general traumatic events were not directly associated with any of the symptom domains. All the childhood adversities were associated with stress reactivity in daily life. Interpersonal adversities (abuse, neglect, bullying, and losses) moderated psychotic-like and/or paranoid reactivity to situational and social stressors, whereas general traumatic events moderated psychotic-like reactivity to situational stress. Also, different interpersonal adversities exacerbated psychotic-like and/or paranoid symptoms in response to distinct social stressors.

**Discussion:**

The present study provides a unique examination of how childhood adversities impact the expression of spectrum symptoms in the real world and lends support to the notion that stress reactivity is a mechanism implicated in the experience of reality distortion in individuals exposed to childhood trauma. Investigating the interplay between childhood experience and current context is relevant for uncovering potential pathways to the extended psychosis phenotype.

## Introduction

There is substantial interest in investigating the etiological relevance of diverse environmental exposures in the development of schizophrenia-spectrum phenotypes [1–3]. Given that mounting evidence supports the hypothesis of etiological continuity between the clinical and subclinical expressions of the schizophrenia spectrum [4–6], focusing on subclinical experiences should enhance the identification of etiological mechanisms while avoiding many of the confounds that complicate the study of clinical samples [7].

Childhood adversity is one environmental exposure that has been widely investigated and shown to be a robust risk factor for schizophrenic phenomenology across a spectrum of severity ranging from schizotypy personality traits to full-blown psychotic disorder [8–10]. In light of this evidence, growing attention is being focused upon elucidating whether particular adverse experiences may contribute to the development of specific symptom domains as well as the mechanistic processes involved [11–13]. These issues are relevant for informing etiological models of symptom formation and may assist the development of prophylactic interventions.

The term childhood adversity has been used in the literature to cover an array of experiences including, among others, different forms of abuse and neglect, bullying victimization, losses, and non-interpersonal events, such as accidents. In general, adverse childhood experiences have been more consistently linked to reality distortion than to negative/disorganized features [10, 14, 15] and available evidence appears to suggest that experiences characterized by an “intention to harm” are more strongly associated with psychotic symptoms than those without intent [16, 17].

It has been proposed that distinct childhood adversities may entail greater risk for different psychosis symptom domains (e.g., [12, 18]). This is based on the hypothesis that different adversities may exert differential influences upon the unfolding of affective and cognitive processes and may thus be expected to show some degree of symptom specificity [12, 19]. However, empirical findings thus far have provided mixed support to this proposition, with some studies indicating that specific childhood adversities are associated with specific psychotic symptoms (e.g., [19, 20]), and others finding no such evidence of specificity (e.g., [17, 21]).

A shortcoming of several previous studies in the field relates to the assessment of childhood adversity. There is limited research employing comprehensive interview measures and many studies either covered a narrow range of adversities or relied on screening measures of adversity [10, 22]. Furthermore, to our knowledge, it has yet to be examined whether the use of different techniques for assessing adverse experiences (interview versus questionnaire) yields similar associations with psychosis symptom domains. Interview measures of life-stress are generally regarded as superior to questionnaires because they allow for probing and clarification of relevant details and minimize biases related to subjective responding [23–25]. However, interviews are often not feasible in large-scale studies due to the labor and time required for their administration [23, 26, 27]. Utilizing both types of measures within the same study may provide insights about the relevance of the assessment methodology in examining the effects of different adversity exposures.

Another relevant issue that has been scarcely investigated concerns the association of different childhood adversities with symptoms assessed using momentary assessment approaches such as the experience sampling methodology (ESM). ESM is a structured diary technique in which individuals are prompted randomly throughout the day to report on their current experiences, such as emotional states, cognitions, and symptoms. This approach offers several advantages compared to traditional assessment procedures, including enhanced ecological validity, minimization of retrospective bias, and the possibility of assessing the context of experiences [28–30]. Notably, ESM has been shown to be a useful tool for examining the clinical and subclinical expressions of the schizophrenia spectrum (e.g., [31–35]) and, given that it captures the phenomenology of symptoms as they unfold in the real world, it may complement current efforts to clarify links between adversity subtypes and psychosis symptom domains.

As regards to mechanistic processes, both theoretical and empirical work suggest that one way in which childhood adversity links to positive psychotic phenomena is through a sensitization process that renders individuals more reactive to subsequent minor stressors in everyday life [36, 37]. Indeed, ESM research has shown that childhood adversity is associated with heightened affective reactions to stress in individuals from the general population [38, 39] and with increased affective and psychotic reactions to stress in patients with psychotic disorder [40].

Although these studies have provided valuable insights regarding the impact of childhood adversity on stress reactivity, there remain issues that require further elucidation. For instance, one previous study focused exclusively on experiences of abuse [38] and the others grouped together experiences of abuse and neglect [39, 40]. Therefore, additional research is needed to examine a broader range of childhood adversities and to determine whether specific adversity subtypes moderate affective and/or symptomatic reactivity to stress. Moreover, these studies focused on event-related and activity-related stress. As such, it is unknown whether similar findings may be observed when focusing on other forms of momentary stress, such as social stress. Drawing from stress-sensitization models, it seems plausible and of notable importance that childhood adversities occurring within the context of interpersonal relationships may increase reactivity to daily life stressors falling in the interpersonal realm.

The present study sought to investigate associations between childhood adversity subtypes and psychosis symptom domains as well as the stress sensitization hypothesis in a nonclinical sample of young adults. Specifically, our aims were to (i) examine the association of different childhood adversities (abuse, neglect, bullying by peers, losses, and general traumatic events) with psychotic-like, paranoid, and negative-like symptoms in daily life; (ii) investigate whether associations of abuse and neglect with daily-life symptoms are consistent across different methods of assessment (interview versus self-report); and (iii) examine the role of different adversity subtypes in moderating affective and symptomatic (psychotic and paranoid) reactivity to different forms of momentary stress (i.e., situational and social).

We expected that childhood adversities would be more consistently linked to psychotic-like and paranoid symptoms than to negative-like symptoms, and that experiences of abuse, neglect, and bullying would be associated with greater risk than experiences with a non-intentional nature (losses) and those occurring outside the relational domain (general traumatic events). Furthermore, we expected that both interview and questionnaire measures of abuse and neglect would show associations with daily life symptoms. However, given that comprehensive interviews that rely on objective definitions of adversity allow for a more precise assessment [24, 26] and may be better suited for delineating more specific models of the effects of adversity exposures (e.g., [41]), we hypothesized that more differentiated patterns of association would emerge with interview-based ratings relative to their questionnaire counterparts. Finally, we hypothesized that interpersonal forms of adversity would be relevant in moderating reactivity to both situational and social stress, whereas general traumatic events would be relevant in moderating reactivity to situational stress.

## Methods

### Ethics Statement

The study was approved by the Ethics Committee of the Universitat Autònoma de Barcelona (Comissió d'Ètica en l'Experimentació Animal i Humana) and conformed to the Helsinki Declaration. The participants had full capacity to consent to participation in research and provided written informed consent prior to taking part in the study.

### Participants

The data were collected as part of an ongoing longitudinal investigation examining psychosis risk and resilience in young adults (PSYRIS-Barcelona). Briefly, usable data were obtained from 547 undergraduate students during mass-screening sessions. Of these, a subset of 339 was invited to take part in a comprehensive assessment (comprising laboratory, questionnaire, interview, and ESM measures) with the aim of assessing 200 individuals. Those invited to participate included 189 with standard scores based upon sample norms of at least 1.0 on questionnaire measures of positive or negative schizotypy, and 150 randomly selected participants with standard scores below 1.0. The objective of the enrichment procedure was to ensure adequate representation of schizotypy in the sample. The final sample for this study consisted of 206 participants (78.6% female) from whom usable self-report, interview, and ESM data were collected. The mean age of the sample was 21.3 (SD = 2.4) years.

### Materials and Procedure

Clinical psychologists and trained advanced graduate students in clinical psychology administered the measures described below.

#### Experiences of abuse and neglect

Participants were administered two measures assessing emotional, physical, and sexual abuse and emotional and physical neglect during childhood and adolescence. The first was a self-report measure, the Childhood Trauma Questionnaire (CTQ) [42]. CTQ items are answered on a 5-point Likert-type scale ranging from “never true” to “very often true” and are added to obtain a score for each type of maltreatment. The second measure was the Interview for Traumatic Events in Childhood (ITEC) [24, 43]. The ITEC is a semi-structured interview in which every item endorsed by the participant is followed by questions covering different parameters including the age of onset, perpetrator(s), frequency, duration, and the level of distress associated with the experience (both at the time and in the present). This information is rated according to predefined answer categories and the objective parameters (act, age, perpetrator, frequency, and duration) are used to calculate composite severity scores for each type of maltreatment. In the present study, indices of childhood abuse and neglect were created from the measures described above. Experiences of abuse and neglect are generally characterized as representing maltreatment by commission and omission, respectively [44]. For both the CTQ and ITEC, sum scores of abuse (sum of physical, emotional, and sexual abuse) and neglect (sum of physical and emotional neglect) were used for analyses.

#### Bullying victimization

Bullying by peers was assessed with questions from the Childhood Experience of Care and Abuse (CECA) [45], a semi-structured, investigator-based interview of childhood experiences. Bullying is scored on a 4-point scale ranging from “marked” to “little/none”, according to specific rating rules and benchmark examples. The analyses used the continuous severity ratings of bullying victimization.

#### Losses and general traumatic events

Participants were administered the general trauma subscale from the Early Trauma Inventory (ETI) [46], a semi-structured interview of childhood trauma. The items in the general trauma subscale cover a wide range of events and do not reflect a unitary construct. Thus, two variables were constructed that assessed: a) experiences of loss and included 5 items: 4 regarding the death of close others (parent or important adult, sibling, friend, and child) and 1 regarding the miscarriage of a child, and b) general traumatic events not occurring in the context of interpersonal relationships and also included 5 items: exposure to a natural disaster, involvement in a serious accident, being the victim of an assault, being the victim of armed robbery, and being held hostage. Scores on these variables were calculated by summing the number of items endorsed, in agreement with previous work (e.g., [47]).

#### ESM assessments

ESM data were collected on personal digital assistants (PDAs), which signaled participants randomly eight times daily (between 10 a.m. and 10 p.m.) for one week to complete brief questionnaires. When signaled by the PDA, participants had 5 minutes to start the questionnaire. After this time window or the completion of the questionnaire, the PDA became inactive until the next signal. The complete list of ESM items can be found in Barrantes-Vidal et al. [31]. Note that all the ESM items used in the current study were answered on 7-point scales from “not at all” to “very much”, with the exception of the social contact item, which was answered dichotomously (alone/with others).

The analyses used ESM measures of symptoms, negative affect, and stress. Following Barrantes-Vidal et al. [31], we created indices of paranoia (2 items: feeling suspicious and mistreated; coefficient α = 0.70) and psychotic-like symptoms (8 items: unusual senses, unusual thoughts, feeling weird, losing control, difficulty controlling thoughts, familiar things seeming strange, hearing/seeing things others could not, and feeling that thoughts/actions are being controlled by someone or something; coefficient α = 0.74), and used the item “Right now I have no thoughts or emotions” as a measure of negative-like symptoms. Negative affect was measured by an index composed of 4 items (feeling anxious, sad, angry, and guilty; coefficient α = 0.83). Situational stress was assessed with the item “My current situation is stressful”. As for social stress, we distinguished between social stress when participants were alone, assessed by the item “I am alone because people do not want to be with me”, and social stress when participants were with others (an index composed of 2 items: not feeling close to others and preferring to be alone; coefficient α = 0.59). In addition, the item asking participants whether they were alone or with others at the time of the signal was used to differentiate the effects of social contact from social stress.

### Statistical Method

Descriptive statistics and correlational analyses were performed on the childhood adversity variables using the Statistical Package for Social Sciences (SPSS). The statistical analyses involving the ESM data were conducted with Mplus 6 [48]. ESM data have a hierarchical structure in which repeated daily life ratings (level 1 data) are nested within participants (level 2 data). Multilevel or hierarchical linear modeling takes into account the nested structure of the data and is a standard approach for the analyses of ESM data [49].

The multilevel analyses examined two types of relations between the childhood adversity variables and experiences rated in daily life. To examine the association of different types of childhood adversities with daily life symptoms, we computed the independent effects of level 2 predictors (adversity variables) on level 1 dependent measures (ESM ratings). To examine whether childhood adversities moderate the momentary association of stress with experiences in daily life, cross-level interactions were conducted. Cross-level interactions test whether the relations between level 1 predictors (e.g., situational stress) and criteria (e.g., paranoia) vary as a function of level 2 variables (e.g., bullying). Following recommendations of Nezlek [49], level 1 predictors were group-mean centered and level 2 predictors were grand-mean centered. Note that level 2 predictors can only be grand-mean centered. Level 1 predictors are group-mean centered to minimize the error from between group (person) mean differences. Data departed from normality in some cases, so parameter estimates were calculated using maximum likelihood estimation with robust standard errors. In addition, level 1 criteria exhibiting substantial skew were treated as categorical.

## Results

Participants completed an average of 40.8 usable ESM questionnaires (SD = 9.1). Descriptive statistics of the childhood adversity variables and their intercorrelations are displayed in Table 1. Following Cohen [50], correlations of self-reported abuse and neglect with their respective interview counterparts were of a large magnitude. Abuse was associated with neglect both within and across measures, with effect sizes ranging from medium to large. Bullying showed a medium correlation with self-reported and interview-based abuse, and a small correlation with self-reported neglect. Losses and general traumatic events were not associated with any of the other adversity variables.

**Table 1:** Descriptive Statistics of Adverse Childhood Experiences and their Intercorrelations (n = 206).

	*M*	*SD*	Range	Abuse CTQ	Neglect CTQ	Abuse ITEC	Neglect ITEC	Bullying	Loss	Traumatic Events
Abuse CTQ	17.89	4.85	15–48	-	*0*.52***	*0*.54***	0.45***	0.33***	0.03	0.00
Neglect CTQ	15.26	4.38	10–32		-	0.43***	*0*.50***	0.21**	0.05	-0.00
Abuse ITEC	5.03	6.16	0–48			-	0.45***	0.42***	0.05	0.11
Neglect ITEC	3.11	5.45	0–30				-	0.09	0.05	0.05
Bullying	0.62	0.93	0–3					-	0.02	0.01
Loss	0.66	0.62	0–3						-	0.11
Traumatic Events	0.32	0.54	0–2							-

Note: CTQ = Childhood Trauma Questionnaire; ITEC = Interview for Traumatic Events in Childhood.

*p<0.05,

**p<0.01,

***p<0.001.

Medium effect sizes (r≥0.30) in bold, large effect sizes (r≥0.50) in bold and italics.

We examined the independent direct effects of childhood adversity on daily life experiences (Table 2). Both self-reported and interview-based abuse and neglect were associated with increased psychotic-like and paranoid symptoms, whereas only self-reported neglect was associated with having no thoughts or emotions. Bullying was associated with increased psychotic-like symptoms. Interview-based and self-reported abuse and neglect, as well as bullying, were associated with increased negative affect. No associations were found with losses or general traumatic events.

**Table 2:** Independent Direct Effects of Adverse Childhood Experiences on Daily Life Outcomes (n = 206).

Level 1 Criterion	Level 2 Predictors						
	Abuse CTQ	Neglect CTQ	Abuse ITEC	Neglect ITEC	Bullying	Loss	Traumatic Events
	γ_01_ (*df* = 204)	γ_01_ (*df* = 204)	γ_01_ (*df* = 204)	γ_01_ (*df* = 204)	γ_01_ (*df* = 204)	γ_01_ (*df* = 204)	γ_01_ (*df* = 204)
	Coefficient (SE)	Coefficient (SE)	Coefficient (SE)	Coefficient (SE)	Coefficient (SE)	Coefficient (SE)	Coefficient (SE)
**Psychosis Spectrum**							
Psychotic-like index	0.009 (0.003)**	0.009 (0.003)**	0.007 (0.002)**	0.006 (0.003)*	0.034 (0.015)*	0.028 (0.019)	0.034 (0.023)
Paranoia index	0.022 (0.008)**	0.023 (0.007)**	0.016 (0.004)***	0.013 (0.006)*	0.038 (0.026)	0.044 (0.038)	0.044 (0.044)
No thoughts/emotions†	-0.002 (0.027)	0.102 (0.039)*	0.007 (0.022)	0.009 (0.034)	0.289 (0.168)	0.177 (0.274)	0.329 (0.280)
**Affect**							
Negative affect index	0.035 (0.008)***	0.027 (0.008)**	0.024 (0.006)***	0.018 (0.008)*	0.113 (0.040)**	0.058 (0.056)	0.078 (0.067)

Note: CTQ = Childhood Trauma Questionnaire; ITEC = Interview for Traumatic Events in Childhood.

^†^Items were run as categorical.

*p<0.05,

**p<0.01,

***p<0.001

Cross-level interaction analyses examined whether childhood adverse experiences moderated the association of social contact and stress appraisals with psychotic-like symptoms, paranoia, and negative affect in daily life (Table 3). As in the analyses of the direct effects, the cross-level effect of each level 2 predictor was examined separately (i.e., level 2 predictors were not entered simultaneously). Each of these analyses computed the association of the level 1 predictor and criterion. Note that the statistical significance of the associations of the level 1 predictor and criterion did not vary across each level 2 predictor, therefore in the table we simply reported the coefficient of the level 1 predictor and criterion for the analysis of CTQ abuse. The results indicated that situational and social stressors were associated with psychotic-like symptoms, paranoia, and negative affect. Being alone at the time of the signal was associated with greater negative affect, but was unrelated to experiencing psychotic-like and paranoid symptoms.

**Table 3:** Cross-Level Interactions of Adverse Childhood Experiences with Daily Life Experiences (n = 206).

Level 1 Criterion	Level 1 Predictors		Level 2 Predictors						
			Abuse CTQ	Neglect CTQ	Abuse ITEC	Neglect ITEC	Bullying	Loss	Traumatic Events
		γ_10_ (*df* = 204)	γ_11_ (*df* = 204)	γ_11_ (*df* = 204)	γ_11_ (*df* = 204)	γ_11_ (*df* = 204)	γ_11_ (*df* = 204)	γ_11_ (*df* = 204)	γ_11_ (*df* = 204)
Indices		Coeff. (SE)	Coeff. (SE)	Coeff. (SE)	Coeff. (SE)	Coeff. (SE)	Coeff. (SE)	Coeff. (SE)	Coeff. (SE)
Psychotic-like	Situation stressful	0.035 (0.004)***	0.001 (0.001)	0.001 (0.001)	0.001 (0.001)	0.002 (0.001)*	0.006 (0.006)	0.015 (0.007)*	0.024 (0.009)**
Psychotic-like	Alone	0.000 (0.006)	-0.001 (0.001)	0.001 (0.001)	-0.001 (0.002)	-0.001 (0.001)	-0.015 (0.006)*	-0.012 (0.011)	0.009 (0.013)
Psychotic-like	Alone b/c unwanted	0.082 (0.019)***	0.001 (0.003)	0.007 (0.005)	-0.002 (0.002)	0.000 (0.003)	0.019 (0.023)	0.011 (0.037)	-0.013 (0.037)
Psychotic-like	Social stress index	0.019 (0.004)***	0.002 (0.001)*	0.003 (0.001)**	0.001 (0.001)	0.002 (0.001)*	0.005 (0.004)	0.008 (0.006)	0.017 (0.009)
Paranoia	Situation stressful	0.078 (0.010)***	0.005 (0.002)*	0.006 (0.002)*	0.003 (0.001)*	0.004 (0.002)	0.029 (0.012)*	0.018 (0.018)	0.035 (0.019)
Paranoia	Alone	-0.008 (0.014)	-0.001 (0.003)	-0.002 (0.003)	0.001 (0.002)	-0.001 (0.003)	0.001 (0.014)	0.004 (0.022)	0.043 (0.029)
Paranoia	Alone b/c unwanted	0.153 (0.050)**	-0.002 (0.009)	0.001 (0.012)	-0.006 (0.006)	0.000 (0.008)	0.039 (0.053)	0.190 (0.078)*	0.017 (0.119)
Paranoia	Social stress index	0.060 (0.011)***	0.005 (0.003)	0.007 (0.003)*	0.007 (0.002)***	0.006 (0.003)*	0.029 (0.013)*	0.037 (0.019)	0.020 (0.023)
Negative affect	Situation stressful	0.214 (0.012)***	0.005 (0.002)**	0.005 (0.002)*	0.002 (0.001)	0.005 (0.002)*	0.015 (0.012)	0.005 (0.022)	0.061 (0.020)**
Negative affect	Alone	-0.047 (0.018)*	-0.002 (0.004)	-0.003 (0.005)	0.000 (0.003)	-0.001 (0.004)	0.012 (0.018)	-0.047 (0.027)	0.015 (0.032)
Negative affect	Alone b/c unwanted	0.176 (0.050)***	-0.002 (0.009)	0.001 (0.014)	-0.002 (0.005)	-0.008 (0.010)	0.075 (0.044)	0.119 (0.084)	0.098 (0.114)
Negative affect	Social stress index	0.109 (0.013)***	0.006 (0.002)**	0.007 (0.003)*	0.004 (0.002)*	0.007 (0.003)*	0.032 (0.015)*	0.025 (0.019)	0.053 (0.024)*

Note: CTQ = Childhood Trauma Questionnaire; ITEC = Interview for Traumatic Events in Childhood.

*p<0.05,

**p<0.01,

***p<0.001

All the childhood adverse experiences were associated with stress-reactivity in daily life. Self-reported abuse moderated the association of social stress when with others with psychotic-like symptoms and that of situational stress with negative affect. Interview-based abuse moderated the association between social stress when with others and paranoia. In addition, both abuse variables moderated the association between situational stress and paranoia and the association between social stress when with others and negative affect. As for experiences of neglect, both self-report and interview ratings moderated the associations of social stress when with others with psychotic-like symptoms, paranoia, and negative affect, along with the association of situational stress with negative affect. Additionally, self-reported neglect moderated the association between situational stress and paranoia, whereas interview-based neglect moderated the association between situational stress and psychotic-like symptoms.

**Fig 1:**
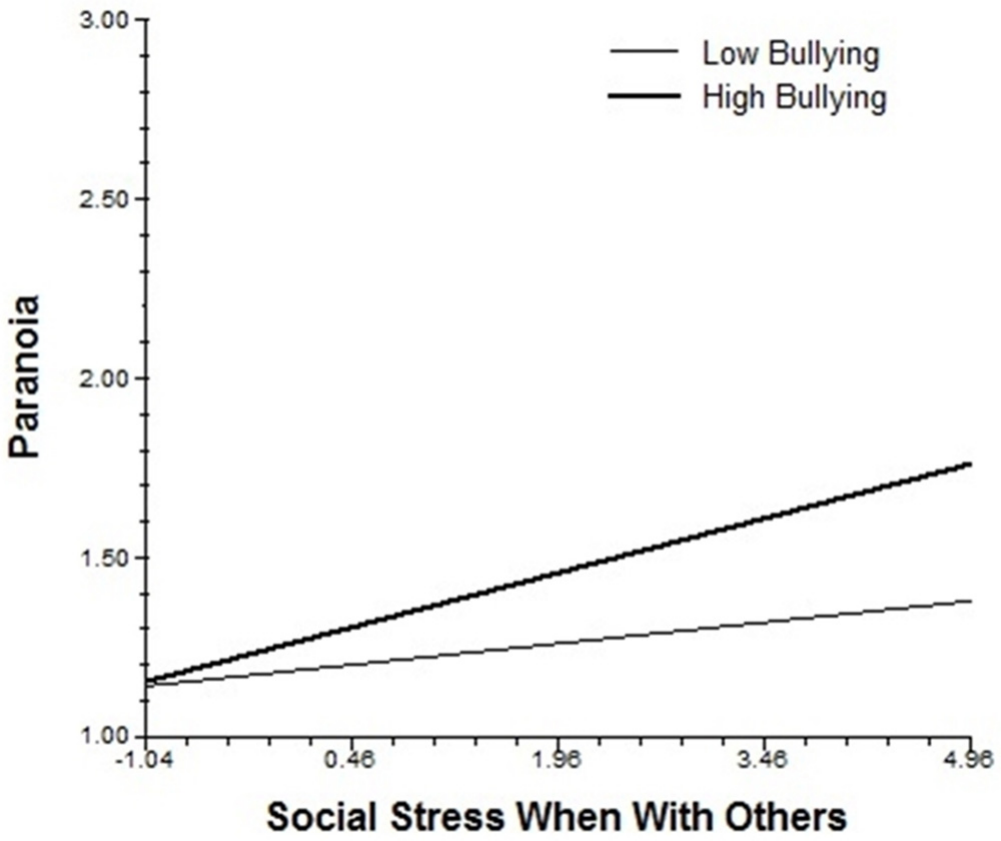
Cross-level interaction of the association of bullying with the slope of social stress when with others and paranoia.

Bullying moderated the slope of social contact and psychotic-like symptoms, such that individuals with higher bullying experienced more psychotic-like symptoms when alone. It also moderated the association of situational stress with paranoia, as well as the associations of social stress when with others with negative affect and paranoia. As seen in Fig 1, when social stress when with others is low, paranoia remains low for everyone; however, as social stress increases, individuals with high levels of bullying experience greater increases in paranoia than those with low levels of bullying.

**Fig 2:**
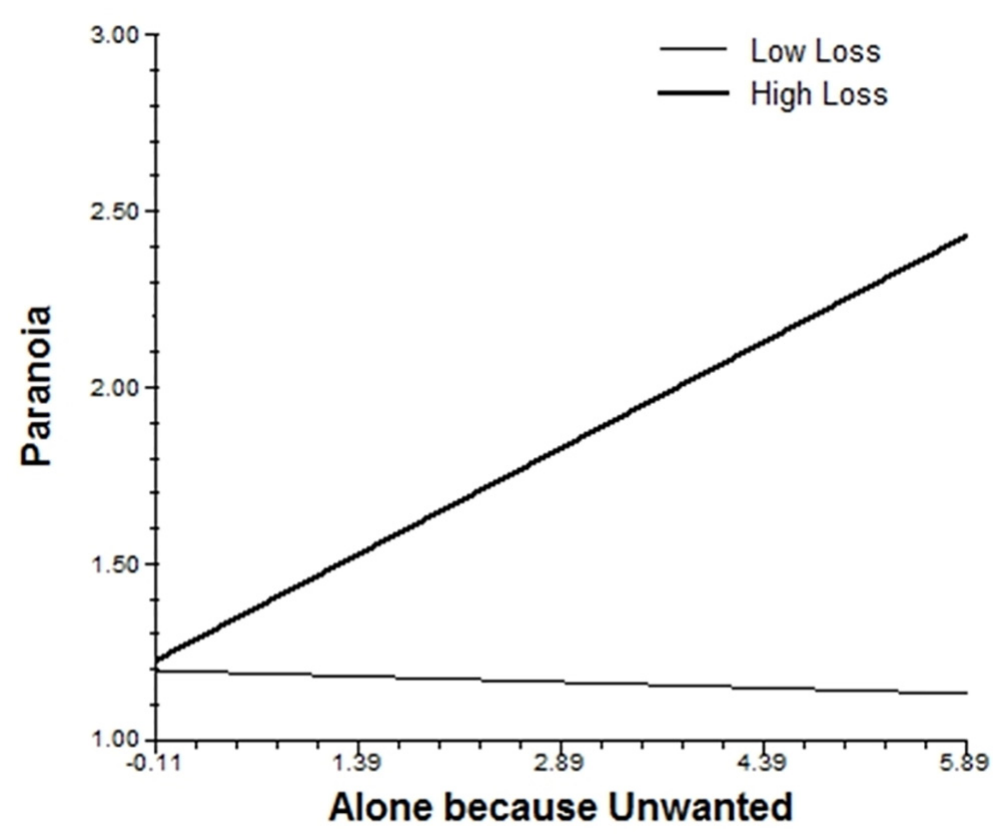
Cross-level interaction of the association of loss with the slope of feeling unwanted by others when alone and paranoia.

Experiences of loss moderated the association between feeling unwanted when alone and paranoia. As displayed in Fig 2, this appraisal was associated with increased paranoid symptoms, but only for individuals with high levels of loss. Finally, both losses and general traumatic events moderated the association of situational stress with psychotic-like symptoms, and general traumatic events also moderated the associations of situational stress and social stress when with others with negative affect.

## Discussion

The present study used ESM to examine the association of different childhood adverse experiences with psychosis spectrum symptoms as well as the stress reactivity hypothesis in a nonclinically ascertained sample of young adults. The study expanded on previous ESM research by measuring a broader range of childhood adversities (using self-report and interview measures) and by assessing affective and symptomatic reactivity to both situational and interpersonal forms of stress. The findings contribute to our understanding of how childhood adversity subtypes impact the expression of spectrum symptoms in the real world and lend further support to the notion that stress reactivity is a mechanism implicated in the experience of reality distortion in individuals exposed to childhood trauma.

The results regarding the adversity-symptom links were in line with our hypotheses. The finding that abuse, neglect, and bullying were associated with positive symptoms is consistent with recent meta-analyses [9, 51], and, importantly, provides evidence that these relations hold for symptoms experienced in the realm of daily life. The only adversity subtype that was associated with having no thoughts or emotions was self-reported neglect. Prior research has provided mixed support for the association between childhood adversity and negative symptoms [14]. However, our results agree with a recent study that used the CTQ in a sample of patients with psychotic disorder, their siblings, and control participants. They found that abuse was particularly relevant for the positive symptom dimension, whereas neglect showed comparable associations with positive and negative symptoms [52]. Experiences of neglect have been associated with deficits in cognitive, social, and emotional domains [53–55], and may play a role in the development of both positive and deficit-like features. We found that losses and general traumatic events were not associated with any of the symptom domains. This resonates with studies in which experiencing the death of a close person [17], being exposed to a natural disaster [56], and having a serious accident [16] showed either weak or no association with psychosis phenotypes. Collectively, the findings indicate that maltreatment (either by commission or omission) and victimization perpetrated by same-age peers are directly linked to the real-life expression of symptoms.

The current study also aimed to add to the literature by investigating whether associations of abuse and neglect with psychosis symptom domains were consistent across interview and self-report methods of assessment. We found that analogous CTQ and ITEC scores were highly related and showed agreement in their associations with psychotic-like and paranoid symptoms. This is a positive finding for the field given that interview measures are frequently not feasible to employ, especially in large-scale investigations [23]. It is worth noting that the abuse and neglect variables showed substantial association, which is consistent with numerous studies indicating that abuse and neglect tend to co-occur [57]; however, this does not preclude that each set of experiences could have certain unique effects in shaping psychological states and maladaptive strategies.

As previously noted, the only difference in the direct effects of the childhood adverse experiences on spectrum symptoms was that the negative-like symptom of diminished thoughts/emotions was associated with self-reported (but not interview-based) neglect. Although the reason for this inconsistency is unclear, it may be related to measurement differences between the two instruments. For instance, in addition to the particular features inherent to questionnaire and interview formats, differences in the wording of neglect items (several CTQ neglect, but not abuse, items are reverse-worded [e.g., “My family was a source of strength and support”], whereas none of the ITEC items are) as well as the distinct ways to quantify maltreatment (the CTQ considers frequency whereas the ITEC considers age, perpetrator, frequency, and duration) may account for this discrepancy.

The results regarding stress reactivity replicate and extend previous ESM research [36–38]. We found that all the adverse experiences investigated were associated with increased reactivity to stress in the flow of daily life. It is interesting to note that although losses and general traumatic events were not directly related to positive symptoms, they were associated with increased symptoms only in interaction with momentary stress. This underscores the importance of examining the joint contribution of distal and momentary stressors to risk for psychotic outcomes.

To our knowledge, this is the first study to investigate whether childhood adversities increase reactivity to stress across situational and social domains. Furthermore, by assessing reactions to both social contact and social stress, the study showed that reactivity was not simply due to being alone or with others, but rather, that it was mostly related to appraisals of social stress. Furthermore, it is worth noting that these findings occurred in a non-clinically ascertained sample of young adults. Thus, childhood adversity may convey risk for subclinical symptoms and stress reactivity in daily life—and these subclinical manifestations may presage the development of schizophrenia-spectrum disorders depending on the complex interaction of genetic, person, and environmental factors across development [58].

Our hypotheses concerning stress reactivity were supported for daily life symptoms. That is, abuse, neglect, bullying, and losses increased psychotic-like and/or paranoid reactivity to situational and social stressors, whereas general traumatic events only increased psychotic-like reactivity to situational stress. Although the findings require replication before drawing firm conclusions, they appear to suggest that only childhood adversities of an interpersonal kind may be relevant for calibrating psychotic-like and paranoid responses to interpersonal stressors. Meanwhile, the findings for negative affect showed a nonspecific pattern of stress-reactivity in relation to the nature of the stressor. Childhood trauma may sensitize individuals to react with increased negative affect, regardless of the specific nature of the distal adversity or the proximal daily life stressor, given the fundamental role of negative affect in the experience of adversity and subsequent re-exposures.

Different interpersonal adversities were found to exacerbate psychotic-like and/or paranoid symptoms in response to distinct social stressors. Specifically, abuse, neglect, and bullying were associated with increased reactivity to social stress when with others, whereas losses were associated with increased reactivity to social stress when alone. In recent years, research findings have converged in supporting a role for negative models/schemas of the self and others in the pathway between interpersonal adversities and psychotic phenomena (e.g., [59–61]). According to attachment theory, early relational experiences shape internal working models (cognitive/affective representations) of the self and others that guide how individuals construe their transactions with the social world [62, 63]. Importantly, internal working models may be activated by appraisals of internal or external threat—and this appraisal process and ensuing regulatory efforts may vary according to an individual’s relational history [63, 64]. Drawing from these notions and prior research, our results may suggest that experiencing social stress *when with others* may be salient for activating negative models in individuals who have experienced neglectful/hostile behavior *from others*. On the other hand, feeling unwanted *when alone* may be salient for activating negative models among those who have experienced loss. The activation of these negative models by specific interpersonal stressors may trigger cognitive and perceptual anomalies leading to the experience of reality distortion.

The strengths of the present work include the comprehensive assessment of childhood adverse experiences, which was conducted using fine-grained interview measures and an extensively used questionnaire, as well as the use of ecologically valid measures of symptoms and stress obtained in real time and on multiple occasions during the course of one week. Limitations of the study include its cross-sectional nature, which precludes conclusions about the causal effects of childhood adversities. Likewise, causal inferences concerning the effects of daily life stressors cannot be definitively drawn, given that predictor and criterion ESM measures were assessed concurrently. In addition, our use of a predominantly female university student sample limits the generalizability of the findings to community samples and clinical populations. At the same time, however, employing a nonclinical sample allows for the assessment of mechanistic processes without the confounding effect of the consequences of a psychotic disorder and minimizes concerns about unreliability of childhood adversity reports due to clinical status. Another consideration is that only one item (having no thoughts or emotions) specifically examined negative symptoms, which may have limited our ability to detect associations between trauma exposures and other negative-like phenomenology. Two issues are noteworthy regarding our assessment of negative symptoms. First, various items in our ESM questionnaire tapped aspects of negative symptoms (e.g., I like what I am doing –reversed- captures anhedonia), but only one (no thoughts or emotions) assessed a markedly deviant experience. These other items tapping negative-like symptoms were designed following recommendations on the assessment of negative symptoms with ESM suggesting that these should be measured in terms of (diminished) experiences of affect, cognition, interest, and social functioning in real life [35]. Naturally, other experiences may contribute to the responses given to these items. In this study, we restricted our comparison to those questions measuring a clear deviant experience, which is the case for all positive symptoms and for the one negative symptom. Secondly, it must be noted that there is a limit to the number of questions that can be included in an ESM protocol, given the frequent and repeated assessments performed during the day. As most evidence has found a more consistent or strong association of adversity exposures with positive rather negative psychotic experiences (e.g., [10, 15]), our questionnaire focused on the latter.

In closing, this study further refines our understanding of how adversity-symptom associations are expressed in real life and the way in which childhood adversity subtypes influence stress reactivity dynamics that may lie on the pathway to the positive dimension of the extended psychosis phenotype. The findings can help inform developmental models of psychosis vulnerability and may have implications for identifying key targets for prophylactic intervention among individuals exposed to childhood adversity.
